# Virtual Environment for Rehabilitation of Upper Distal Limb Using a Haptic Device with Adaptive Impedance Control and Neural Compensation: A Preliminary Proposal

**DOI:** 10.3390/s25195964

**Published:** 2025-09-25

**Authors:** Yahel Cortés-García, Yukio Rosales-Luengas, Saul J. Rangel-Popoca, Sergio Salazar, Xiaoou Li, Rogelio Lozano

**Affiliations:** Department of Research and Multidisciplinary Studies, Center for Research and Advanced Studies, Mexico City 07360, Mexico; yaheljesus.cortes@cinvestav.mx (Y.C.-G.); yrosales@cinvestav.mx (Y.R.-L.); saul.rangel@cinvestav.mx (S.J.R.-P.); sesalazar@cinvestav.mx (S.S.); xiaoou.li@cinvestav.mx (X.L.)

**Keywords:** Novint Falcon, haptic device, rehabilitation, impedance control, virtual environment

## Abstract

This research presents a preliminary proposal for a rehabilitation exercise aimed at patients with muscle weakness in the distal upper limb. A virtual environment was developed, where the user engages in a rehabilitation activity focused on rehabilitating the pinch grip. The goal is to strengthen the patient’s grasp and reduce muscle weakness. The virtual environment was designed as a video game in order to generate greater interest and encourage patients to adhere to their rehabilitation activities. This virtual game utilizes the haptic device Novint Falcon for the interaction with the environment. This preliminary work implements an impedance control with neural compensation; the control strategy produces signals to adapt the force exerted by the patient, with the goal that the device can give a force of the same magnitude but in the opposite direction. Consequently, regardless of the patient’s initial strength, the device will always deliver an assistive force to guide the patient along a desired trajectory. Initial experimental results with the proposed virtual-haptic rehabilitation system are presented, indicating the feasibility of the approach; however, further studies are required to validate its clinical effectiveness.

## 1. Introduction

Cerebrovascular Disease (CVD) is a major social, economic and health problem due to the great disability it generates. This disease can be defined as a disorder in which an area of the brain is temporarily or permanently affected by ischemia or hemorrhage, involving one or more cerebral blood vessels affected by a pathological process [1].

There are different ways to prevent and treat this condition; however, when the disease has already advanced and the patient is left with sequelae, it is necessary to develop certain physiotherapy activities as soon as possible. This is performed with the purpose of preventing possible complications or maintaining organic functions. If the patient’s condition is advanced, performing physiotherapy activities will help restore certain lost functional capacities, as well as adapt residual functions [2].

Rehabilitation activities should be based on daily tasks, with or without tools. The goal of completing activities of daily living is for the patient, at the end of the rehabilitation, to be reintegrated into society without needing to depend on someone else. As previously mentioned, rehabilitation sessions can sometimes be supported by devices that help perform the activities in a more practical manner, as explained in [3,4], where the devices are responsible for reactivating the person’s motor skills in object handling activities. These devices can be elements previously validated by the medical field; they may also be proposals for modified or innovative devices to perform a specific rehabilitation activity. This is the case in [5], where the authors use a device to help patients with hemiplegia. In this research, they decided to take a stationary bicycle as a reference and adapt it to perform specific rehabilitation exercises. On the other hand, rehabilitation devices have also been developed to be more economical than the existing robots, such as in [6,7], where the authors use 3D-printing to facilitate the construction of a device and reduce costs, or in [8], where the authors use low-cost development boards, such as Arduino UNO.

Within these rehabilitation devices, there are assistant robots for passive rehabilitation, which are programmed to perform a specific activity. In these cases, the specialist is responsible for ensuring that the robot executes the established instructions correctly. As the rehabilitation progresses, it is necessary for the user to actively collaborate with the device, so that the patient can recover not only the movement but also the strength necessary to perform daily life activities [9,10,11].

Sometimes, it is not only necessary to have the physical elements to develop the rehabilitation sessions; there are situations where the patient must remain motivated so as not to interrupt their rehabilitation. For example, in [12], the authors propose using the NAO robot so that children with hemiplegia could see the movements they had to imitate reflected. Another way to maintain a playful interest in rehabilitation activities is by involving virtual environments [13]. This refers to an artificial generated space in which the patient can engage with a different activity. The best known example is video games; however, with the advancement of technology, virtual reality can also simulate that the patient is in a real-world environment or can recreate certain activities that currently present complications. For example, in [14], the authors worked on the rehabilitation of patients with hemiplegia, in which, through a simulation of their healthy arm, they can see a reflection of how the exact movement of the opposite arm should be, which helps the patient visualize the movements that they can achieve.

Another way to recreate a more interesting rehabilitation environment is through the use of a haptic system. This can be defined as a device that allows one to touch, feel and interact with an object in a remote environment [15]. This ability allows the patient to experience the sensation of interacting with their surroundings without needing to have the manipulative objects physically present. Because of this, different companies are dedicated to creating their own haptic devices, although not all are directly focused on rehabilitation. These devices are, most of the time, designed to interact with virtual environments—the best known are video games. However, due to their haptic capabilities, several studies have been undertaken with the purpose of using them in the rehabilitation field [16].

These devices can be divided into two types [17]; on one side, there are wearable exoskeletons, which allow for more precise manipulation, giving better control over the movements that are to be simulated. These exoskeletons have the characteristic that they can work exclusively with the hand segment or involve the whole arm [18]. These devices are designed to be lightweight so that the patient does not have complications when using them, as explained in [19,20], where the authors designed their own devices to be as light as possible, with the purpose of adapting to different trajectories in the rehabilitation activities. Furthermore, these devices must be able to fully simulate hand function, which includes the forces with which people normally work, as well as accommodating the anatomy of different users. For this, in [21], the authors proposed a hybrid rigid-flexible-soft mechanism, demonstrating that a device can combine the precision of rigid links with the adaptability of soft components. This design enables the device to develop physiological pinch trajectories while maintaining constant forces at the fingertips during training, demonstrating stability and adaptability.

On the other hand, there are end-effector devices. The difference is that these devices can provide better feedback signals; however, they are completely static, as is the case in [22], where the authors developed a device that allows performing several trajectories, but only within the range for which it was designed. Among the best-known end-effector devices are the Phantom Series, Omega and Delta Series and the Novint Falcon. Each of these devices was born from the premise of having an immersive interaction so that the person can see their actions reflected in a virtual environment. Also, these devices are cheaper than those that focus directly on rehabilitation [23].

The device used in this research is the Novint Falcon. This device was created to be a video game controller that can feed back the force perceived by the character being controlled. It has a spherical end-effector and four buttons that perform different functions depending on the video game being played. Given its haptic characteristics, this device has been explored to modify the end-effector. For example, in [24], the authors designed a complement to be installed on the end-effector to make it easier to press the buttons and perform the rehabilitation exercises. On the other hand, there have also been cases where the end-effector was modified entirely, as in [25], where the authors utilized various modifications in order to obtain movements which the device cannot perform naturally.

These modifications to the device take advantage of its haptic feature to work in different ways. For example, in [26], the authors create a simulation for the patient to cross a virtual pipe; the haptic feedback serves its function by allowing the user to perceive the edges of this pipe, forcing the patient to follow the internal path. It is also possible to simulate different maze style boards or boards with obstacles in order to evaluate the performance of patients, as explained in [27,28]. Another innovation that has been applied to this device is working with two elements simultaneously. In these cases, the rehabilitation tasks become more specific, as in the case of [29], where the authors focused on strengthening the pinch movement.

The main contribution of this article is the proposal of a rehabilitation exercise using the Novint Falcon device. The purpose of the exercise is to recover muscle strength in the grip movement, known as the pinch grip, allowing patients who have suffered muscle weakness due to CVD to regain the ability to manipulate objects. This activity will have the characteristic that, as rehabilitation progresses, the pinch movement will require greater force to be performed. This force increment will be managed through impedance control, where the constants will simulate a spring and its damping and will be predicted by a neuronal network. It is important to note that this work represents an initial conceptual design and preliminary implementation, and has not yet been clinically validated.

This article is organized as follows: Section 2 explains the device and its working environment, as well as its kinematic and dynamic models. Section 3 explains the control applied to the device. Section 4 presents the experimental results, and Section 5 concludes the research.

## 2. Materials and Methods

### 2.1. Rehabilitation Exercise

The Novint Falcon device was created, by Novint Technologies Inc., Albuquerque, NM, USA, to be used as a video game controller. The innovative feature of this device is its haptic ability, which enables the patient to feel a feedback of force by the video games. This haptic ability is used in order to develop rehabilitation exercises which demand a specific force to be applied by the patient. This research sought to replicate the pinch movement; this movement is essential for humans, as it involves the capacity to hold or grab objects by applying a certain force. Initially, this exercise is carried out by means of handling a rubber ball; the exercise consists of opening and closing the fingers, and repeating this movement during a period determined by a specialist. The advantage of implementing the pinch exercise on a haptic device is that it allows the patient to experience variable grip forces without the necessity to change tools or to use additional rehabilitation instruments. A further advantage is that with impedance control, the system measures the position and force applied by the patient to adapt the resistive forces in real time, allowing the patient to work gradually in order to progress in their rehabilitation. To support this, a virtual environment was created, the purpose of which is to maintain motivation and adherence to the rehabilitation sessions. In addition to provision of the virtual environment, the final effector of the device was modified, along with the physical setup in which the rehabilitation tasks are performed.

### 2.2. Virtual Environment

The virtual environment was created using IDE Visual Studio 2022 Version 17.14.0, and the C++ programming language. It was developed in this way to ensure compatibility with Chai3D, a repository of different open source programs. These libraries were also selected due to their modular architecture, which allows extending functions within the code in the event that there is a need to add further activities; this is highly beneficial for rehabilitation cases where patients change or exhibit some improvement.

**Figure 1:**
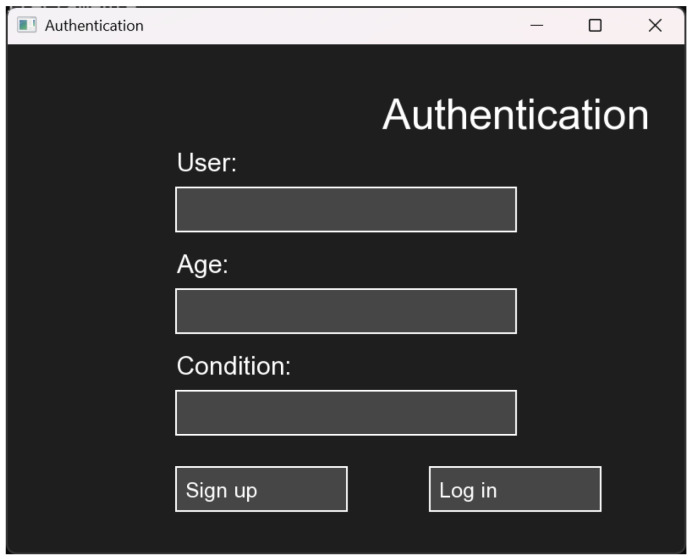
Patient registration.

The virtual environment has a start window for patient registration, as can be seen in Figure 1. The data requested at the outset are the patient’s name, age and any condition in the hand—this is done to enable a record of patients to be kept.

**Figure 2:**
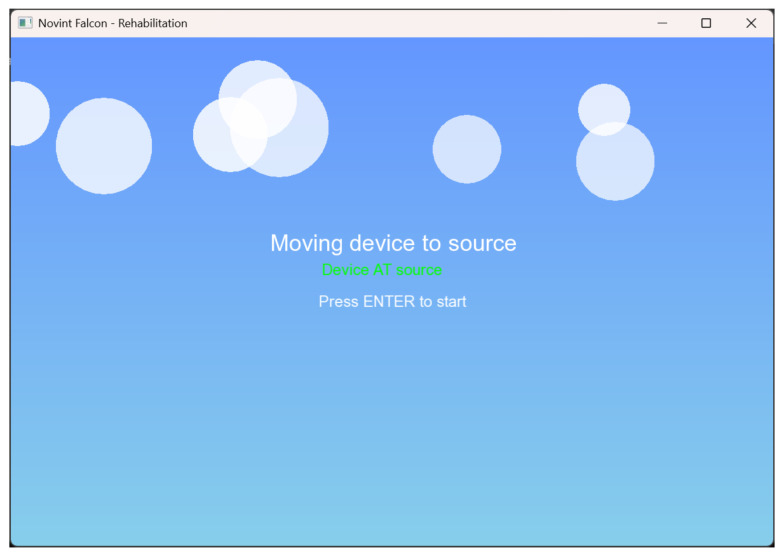
Positioning at the source.

Once the patient is registered, the Novint Falcon automatically positions itself at the source, with the objective that each session starts in a standardized position, eliminating possible errors and ensuring repeatability. This involves use of a waiting window which allows the user to advance, as shown in Figure 2.

**Figure 3:**
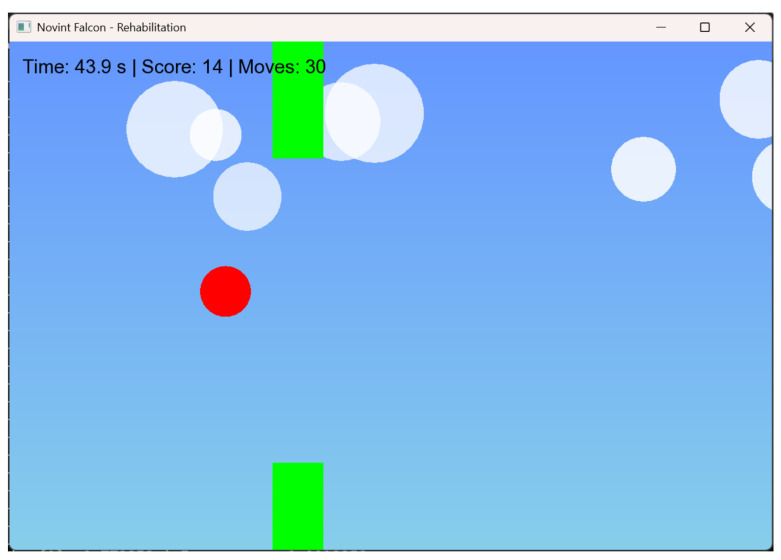
Game frame in play.

Once inside the game, a red circle is presented which is the character, which moves with a small jump each time the user makes a pinching movement on the device (moving the final effector on the Z-axis), as shown in Figure 3. The user must avoid the obstacles (represented as green rectangular) that appear in the video game; if the patient avoids impacting with the obstacles, the effort to perform the movement increases, and in the event of impacting with an obstacle, the game will end.

A side-scrolling dynamic was chosen because the appearance of obstacles encourages patients to perform repetitive pinch movements with the aim of avoiding them. This approach aligns with research reported in [30], where the authors demonstrated that using a video game leads to patients performing more repetitions per rehabilitation session compared to traditional exercises. Consequently, exercises that generate more repetitions lead to better results. Another reason for choosing this dynamic was its ability to generate obstacles randomly within the visual range—as a result, each repetition and session is unique, helping to keep the patient engaged and motivated in each rehabilitation session. At the end of the session time, a window with the number of obstacles overcome is shown. The main purpose of presenting these data is to provide a quantifiable goal for the patient to achieve each session. In this way, the patient is encouraged to perform more repetitions of the pinch movement each session.

**Figure 4:**
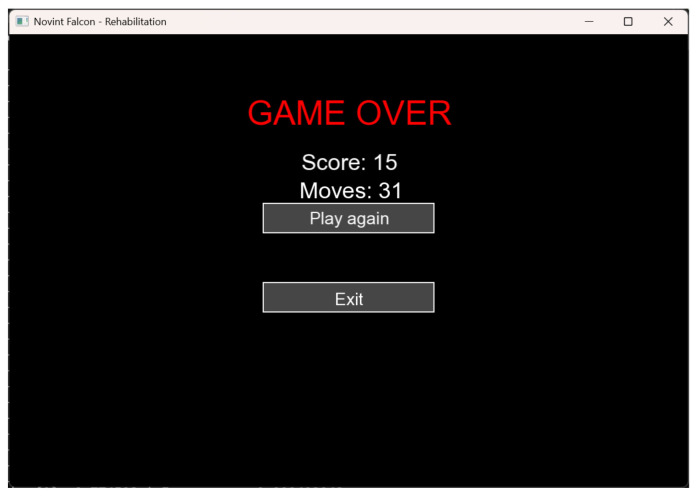
End of game window.

This window also allows restarting the level or exiting to enable registration or logging in of a new patient. These features can be seen in Figure 4.

When the session ends, patient data, such as strength, position, obstacles overcome, and number of repetitions of a movement, are recorded, among others. These data can then be analyzed to determine if the exercise is helping the patient with their rehabilitation.

### 2.3. Changes to the Environment

**Figure 5:**
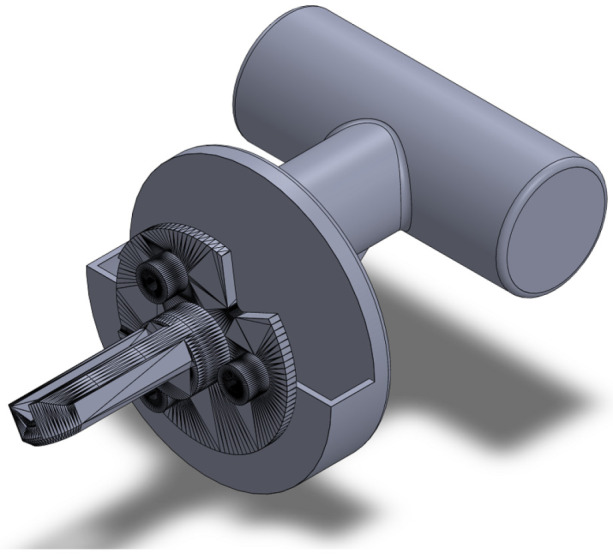
Three-dimensional design of the final effector.

As already mentioned above, in order to enable the movement to be performed, it was necessary to modify, in its entirety, the final effector. The design was created using the SolidWorks 2022 SP0.0 and then printed in 3D in PETG material. This design is shown in Figure 5.

**Figure 6:**
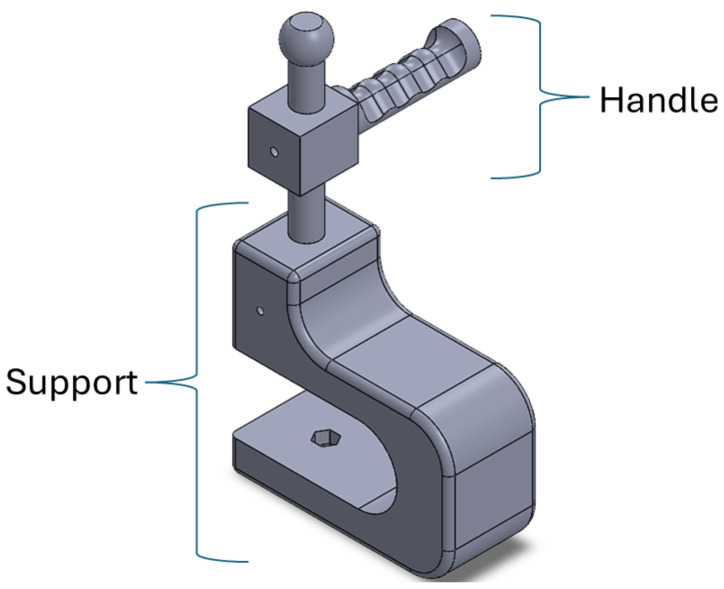
Design of the combined accessories.

In addition to this element, it was also necessary to develop a couple of complements so that the movement of the pinch is carried out in the correct way. In Figure 6, the accessories which had to be printed are illustrated. The purpose of the element denoted handle is to provide the patient with a surface that enables performing the pinch movement. The support element is attached to a surface such as a table, with the objective of maintaining a fixed space in which to perform the movement. An advantage of the complements is that the handle can rotate, and can therefore be adapted for any limb requiring rehabilitation.

**Figure 7:**
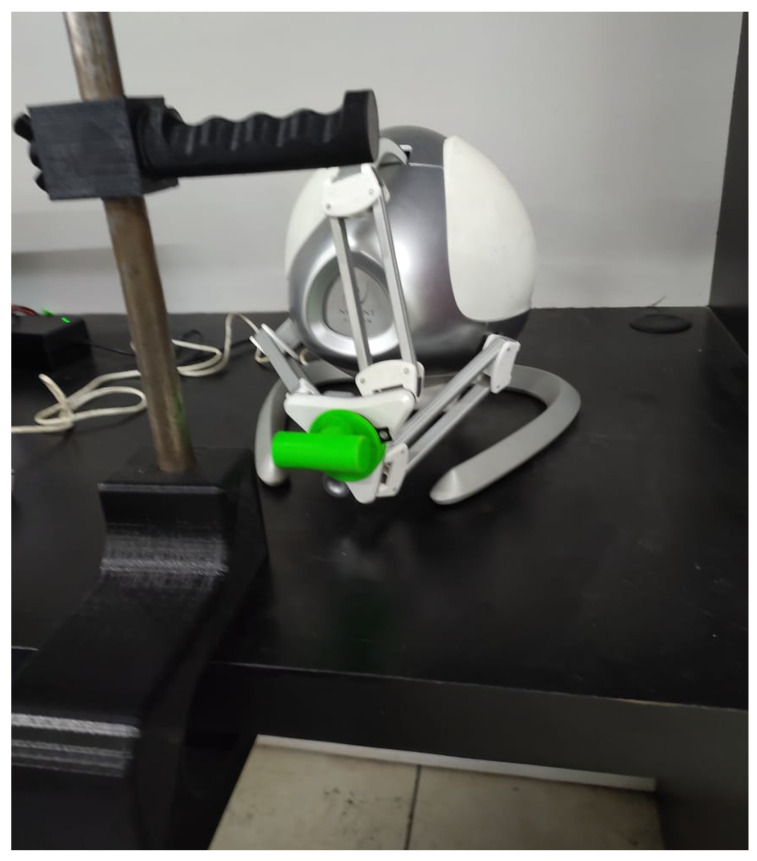
Complete proposed prototype.

With the 3D-printed parts in Figure 5 and Figure 6, it is possible to perform the movement required for rehabilitation. Figure 7 shows the proposed prototype for the rehabilitation exercise.

**Figure 8:**
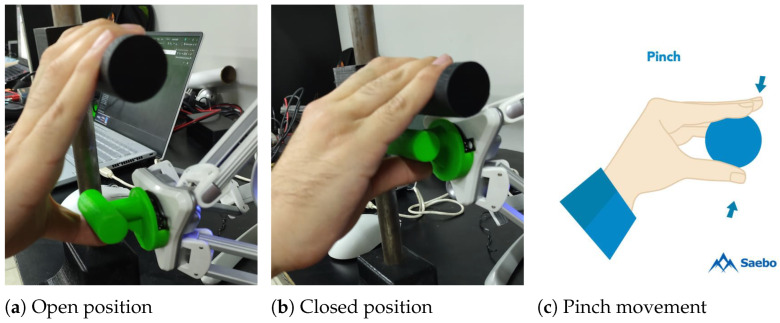
Example of pinch movement. (**a**) Represents the maximum opening of the hand, which can be modified to suit different patients. (**b**) Represents the minimum closure of the hand to perform the movement. (**c**) Represents the traditional rehabilitation exercise which inspired the proposed system [31].

Figure 8 illustrates the movements applied in the proposed rehabilitation system. The Novint Falcon interprets the movements of the user’s hand in terms of linear displacements within the workspace. This parallel configuration allows control of the Z-axis, ensuring that the pinch exercise can be reproduced while taking account of force feedback.

The proposed exercise consists of the user starting with their hand in an open position, placing the thumb under the end-effector and the remaining fingers on the handler. The idea is for the user to close their hand, simulating the pinch movement, as shown in Figure 8. Since no prior calibration is required, simply running the game is sufficient—during the pinch movement, the device will adapt the resistance depending on the impedance controller parameters and the force applied by the patient when closing the hand. Each time this movement is performed, interaction with the virtual environment will occur.

### 2.4. Kinematic Model

In this section, both the kinematic and dynamic models are presented.

**Figure 9:**
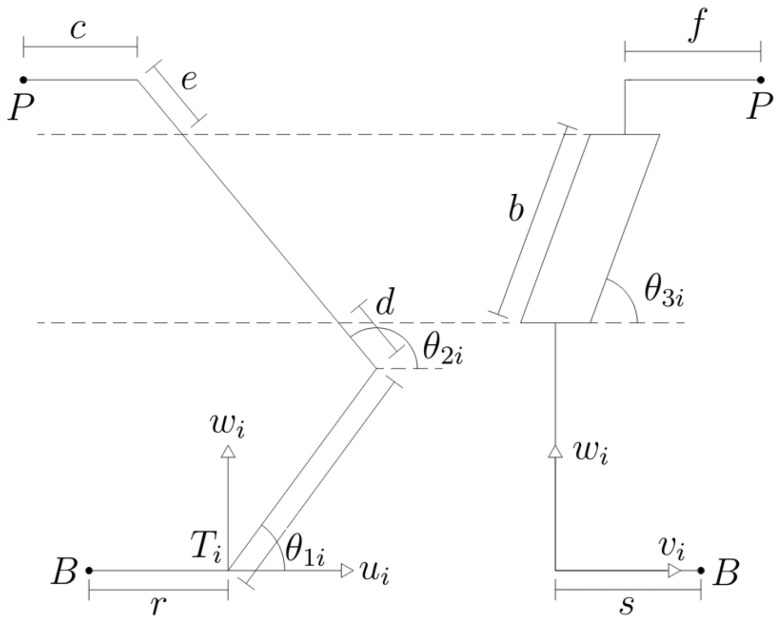
Free-body diagram of the Novint Falcon [32].

The kinematic model of this system is represented by the free-body diagram shown in Figure 9.

From the diagram in Figure 9, the sum of forces for the joints can be derived. These are represented as follows:

(1) $$\begin{array}{cc} p_{\mathrm{ui}} & =a\cos\left(\theta_{1i}\right)-c+\left[d+e+b\sin\left(\theta_{3i}\right)\right]\cos\left(\theta_{2i}\right) \end{array}$$

(2) $$\begin{array}{cc} p_{\mathrm{vi}} & =b\cos\left(\theta_{3i}\right)+f \end{array}$$

(3) $$\begin{array}{cc} p_{\mathrm{wi}} & =a\sin\left(\theta_{1i}\right)+\left[d+e+b\sin\left(\theta_{3i}\right)\right]\sin\left(\theta_{2i}\right)\; \end{array}$$

To determine each of the angles, it is necessary to solve the above equations. The following expressions can then be obtained:

(4) $$\begin{array}{cc} \; & \theta_{1i}=2\arctan\left(\alpha_{i}\right)=\theta_{1i} \end{array}$$

(5) $$\begin{array}{cc} \; & \theta_{2i}=\arcsin\left(\frac{p_{wi}-a\sin\left(\theta_{1i}\right)}{d+e+b\sin\left(\theta_{3i}\right)}\right) \end{array}$$

(6) $$\begin{array}{cc} \; & \theta_{3i}=\arccos\left(\frac{p_{vi}-f}{b}\right)\; \end{array}$$

Having derived these equations, it is necessary to determine the Jacobian matrix. This matrix represents the transformation of the velocity of the final effector ($\dot{x}$) to the velocity of the actuated joints ($\dot{q}$), which are located at the base of the robot. In order to obtain this matrix, first, the relation of the chain closed kinematics must be obtained. This relation is expressed as follows:

(7) $$\begin{array}{c} \bar{w_{1i}}\times \bar{TB_{i}}={\dot{P}}_{T_{i}}+\bar{w_{3i}}\times \bar{DC_{i}}+\bar{w_{2i}}\times \left(\bar{ED_{i}}+\bar{CB_{i}}\right) \end{array}$$

where ${\dot{P}}_{T_{i}}={\left[{\dot{p}}_{\mathrm{ui}},{\dot{p}}_{\mathrm{vi}},{\dot{p}}_{\mathrm{wi}}\right]}^{T}$, $\bar{TB_{i}},\;\bar{DC_{i}},\;\bar{ED_{i}},\;\bar{CB_{i}}$ are the link position vectors, and $w_{\mathrm{ni}}$ is the angular velocity of the n-th link defined as

(8) $$\begin{array}{c} \bar{w_{1i}}=\left[\begin{array}{c} 0\\ -{\dot{\theta }}_{1i}\\ 0 \end{array}\right],\;\;\bar{w_{2i}}=\left[\begin{array}{c} 0\\ -{\dot{\theta }}_{2i}\\ 0 \end{array}\right],\;\;\bar{w_{3i}}=\left[\begin{array}{c} {\dot{\theta }}_{3i}\sin\left(\theta_{2i}\right)\\ -{\dot{\theta }}_{2i}\\ -{\dot{\theta }}_{3i}\cos\left(\theta_{2i}\right) \end{array}\right] \end{array}$$

Solving Equation (7) gives the relation of the chain closed kinematics in terms of link parameters and joint angles, expressed as follows:

(9) $$\begin{array}{c} \left[\begin{array}{c} -a{\dot{\theta }}_{1i}\sin\left(\theta_{1i}\right)\\ 0\\ a{\dot{\theta }}_{1i}\cos\left(\theta_{1i}\right) \end{array}\right]=\left[\begin{array}{c} {\dot{p}}_{\mathrm{ui}}-b{\dot{\theta }}_{3i}\cos\left(\theta_{2i}\right)\cos\theta_{3i}+{\dot{\theta }}_{2i}\sin\left(\theta_{2i}\right)\left[d+e+b\sin\left(\theta_{3i}\right)\right]\\ {\dot{p}}_{\mathrm{vi}}+b{\dot{\theta }}_{3i}\sin\left(\theta_{3i}\right)\\ {\dot{p}}_{\mathrm{wi}}-b{\dot{\theta }}_{3i}\sin\left(\theta_{2i}\right)\cos\theta_{3i}-{\dot{\theta }}_{2i}\cos\left(\theta_{2i}\right)\left[d+e+b\sin\left(\theta_{3i}\right)\right] \end{array}\right] \end{array}$$

Equation (9) can be solved to obtain each of the angular velocities. Taking the angular velocity ${\dot{\theta }}_{1i}$, the following is obtained:

(10) $$\begin{array}{c} {\dot{\theta }}_{1i}=\frac{{\dot{p}}_{\mathrm{ui}}\cos\left(\theta_{2i}\right)+{\dot{p}}_{\mathrm{wi}}\sin\left(\theta_{2i}\right)+{\dot{p}}_{\mathrm{vi}}\cot\left(\theta_{3i}\right)}{a\sin\left(\theta_{2i}-\theta_{1i}\right)} \end{array}$$

The Jacobian matrix is determined by partially deriving Equation (10), recalling that this equation is obtained in the reference frame of each limb; therefore, it is necessary to transform it to the base frame, thus obtaining the following Jacobian matrix:

(11) $$\begin{array}{c} J=\left[\begin{array}{ccc} \frac{\cos\left(\phi_{1}\right)\cos\left(\theta_{21}\right)-\sin\left(\phi_{1}\right)\cot\left(\theta_{31}\right)}{a\sin\left(\theta_{21}-\theta_{11}\right)} & \frac{\sin\left(\phi_{1}\right)\cos\left(\theta_{21}\right)-\cos\left(\phi_{1}\right)\cot\left(\theta_{31}\right)}{a\sin\left(\theta_{21}-\theta_{11}\right)} & \frac{\sin\left(\theta_{21}\right)}{a\sin\left(\theta_{21}-\theta_{11}\right)}\\ \frac{\cos\left(\phi_{2}\right)\cos\left(\theta_{22}\right)-\sin\left(\phi_{2}\right)\cot\left(\theta_{32}\right)}{a\sin\left(\theta_{22}-\theta_{12}\right)} & \frac{\sin\left(\phi_{2}\right)\cos\left(\theta_{22}\right)-\cos\left(\phi_{2}\right)\cot\left(\theta_{32}\right)}{a\sin\left(\theta_{22}-\theta_{12}\right)} & \frac{\sin\left(\theta_{22}\right)}{a\sin\left(\theta_{22}-\theta_{12}\right)}\\ \frac{\cos\left(\phi_{3}\right)\cos\left(\theta_{23}\right)-\sin\left(\phi_{3}\right)\cot\left(\theta_{33}\right)}{a\sin\left(\theta_{23}-\theta_{13}\right)} & \frac{\sin\left(\phi_{3}\right)\cos\left(\theta_{23}\right)-\cos\left(\phi_{3}\right)\cot\left(\theta_{33}\right)}{a\sin\left(\theta_{23}-\theta_{13}\right)} & \frac{\sin\left(\theta_{23}\right)}{a\sin\left(\theta_{23}-\theta_{13}\right)} \end{array}\right] \end{array}$$

### 2.5. Dynamic Model

With the Jacobian matrix, the relation between the velocities of the final effector and the joints is realized. The first formula to be obtained represents the forces applied in the actuated articulation (rotational and translational inertia), expressed as

(12) $$\begin{array}{c} M_{\mathrm{AIi}}=I_{A}{\ddot{\theta }}_{1i}+{\tilde{\tau }}_{i}\; \end{array}$$

where $\theta_{i}$ is the angular position of the joints with i∈{1,2,3}, $M_{\mathrm{AIi}}\in R^{3\times 1}$ is the sum of the moments in the articulation, $I_{A}\in R^{3\times 1}$ is the inertia moment, and ${\tilde{\tau }}_{i}\in R^{3\times 1}$ are the inertial loads.

This, in turn, provides the relation for $I_{A}$ y $\tilde{\tau }$

(13) $$\begin{array}{c} I_{A}=I_{r}+\frac{1}{3}m_{a}a^{2}+m_{b}a^{2}\; \end{array}$$

where $I_{r}$ is the moment of inertia of the rotor, $m_{a}$ is the mass of the link, $m_{b}$ is the mass of one of the connecting rods of the parallelogram, and *a* is the length link; all the elements are constant and by the structure of the device, all links have the same value.

On the other hand, $\tilde{\tau }$ is expressed as

(14) $$\begin{array}{c} \tilde{\tau }={\left(J^{T}\right)}^{-1}\left(3m_{b}+m_{c}\right){\ddot{P}}_{B}\; \end{array}$$

where $m_{c}$ is the mass of the mobile platform which is constant, $J\in R^{3\times 3}$ is the Jacobian matrix, and ${\ddot{P}}_{B}\in R^{3\times 1}$ is the acceleration of the mobile platform, which relation is obtained by

(15) $$\begin{array}{c} {\ddot{P}}_{B}=J^{-1}\left[\begin{array}{c} {\ddot{\theta }}_{11}\\ {\ddot{\theta }}_{12}\\ {\ddot{\theta }}_{13} \end{array}\right]+\frac{d}{dt}\left(J^{-1}\right)\left[\begin{array}{c} {\dot{\theta }}_{11}\\ {\dot{\theta }}_{12}\\ {\dot{\theta }}_{13} \end{array}\right] \end{array}$$

The second motion equation is obtained by adding the movements applied in the passive joint, as follows:

(16) $$\begin{array}{cc} \left[\begin{array}{c} M_{{\mathrm{AT}}_{1}}\\ M_{{\mathrm{AT}}_{2}}\\ M_{{\mathrm{AT}}_{3}} \end{array}\right]=\left[\begin{array}{c} \tau_{1}\\ \tau_{2}\\ \tau_{3} \end{array}\right] & +\frac{1}{2}am_{a}g\left[\begin{array}{c} \sin\left(\phi_{1}\right)\sin\left(\theta_{11}\right)\\ \sin\left(\phi_{2}\right)\sin\left(\theta_{12}\right)\\ \sin\left(\phi_{3}\right)\sin\left(\theta_{13}\right) \end{array}\right]+am_{b}g\left[\begin{array}{c} \sin\left(\phi_{1}\right)\sin\left(\theta_{11}\right)\\ \sin\left(\phi_{2}\right)\sin\left(\theta_{12}\right)\\ \sin\left(\phi_{3}\right)\sin\left(\theta_{13}\right) \end{array}\right]\\ \; & +{\left(J^{T}\right)}^{-1}m\left[\begin{array}{c} 0\\ -g\\ 0 \end{array}\right]-b_{A}\left[\begin{array}{c} {\dot{\theta }}_{11}\\ {\dot{\theta }}_{12}\\ {\dot{\theta }}_{13} \end{array}\right] \end{array}$$

where $\tau_{i}$ is the moment applied by the actuator to the extremity, $m=3m_{b}+m_{c}$, $b_{A}$ is the viscous coefficient of friction, and *g* is the gravity. $m,\;b_{A},\;g$ are constants.

By means of Newton’s Second Law, the moments in the articulation are related by

(17) $$\begin{array}{c} \left[\begin{array}{c} M_{{\mathrm{AT}}_{1}}\\ M_{{\mathrm{AT}}_{2}}\\ M_{{\mathrm{AT}}_{3}} \end{array}\right]=\left[\begin{array}{c} M_{{\mathrm{AI}}_{1}}\\ M_{{\mathrm{AI}}_{2}}\\ M_{{\mathrm{AI}}_{3}} \end{array}\right] \end{array}$$

In other words, it has

(18) $$\begin{array}{cc} \left[\begin{array}{c} \tau_{1}\\ \tau_{2}\\ \tau_{3} \end{array}\right]=-ag\left(\frac{1}{2}m_{a}+m_{b}\right) & \left[\begin{array}{c} \sin\left(\phi_{1}\right)\sin\left(\theta_{11}\right)\\ \sin\left(\phi_{2}\right)\sin\left(\theta_{12}\right)\\ \sin\left(\phi_{3}\right)\sin\left(\theta_{13}\right) \end{array}\right]-{\left(J^{T}\right)}^{-1}m\left[\begin{array}{c} 0\\ -g\\ 0 \end{array}\right]+b_{A}\left[\begin{array}{c} {\dot{\theta }}_{11}\\ {\dot{\theta }}_{12}\\ {\dot{\theta }}_{13} \end{array}\right]\\ \; & +I_{A}\left[\begin{array}{c} {\ddot{\theta }}_{11}\\ {\ddot{\theta }}_{12}\\ {\ddot{\theta }}_{13} \end{array}\right]+{\left(J^{T}\right)}^{-1}m{\ddot{P}}_{B} \end{array}$$

Grouping the terms of Equation (18), the representation of the general equation of motion can be obtained

(19) $$\begin{array}{c} \tau =H\left(q\right)\ddot{q}+C\left(q,\dot{q}\right)\dot{q}+D\dot{q}+g\left(q\right)\; \end{array}$$

where $H\left(q\right)\in R^{3\times 3}$ is the inertia matrix, $C\left(q,\dot{q}\right)\in R^{3\times 3}$ is the Coriolis matrix, $D\in R^{3\times 3}$ is the friction force matrix, and $g\left(q\right)\in R^{3\times 1}$ is the gravity vector. These matrices are given by

(20) $$\begin{array}{cc} H(q) & =I_{A}I+{\left(J^{T}\right)}^{-1}mJ^{-1} \end{array}$$

(21) $$\begin{array}{cc} C(q,\dot{q}) & ={\left(J^{T}\right)}^{-1}m\frac{d}{dt}\left(J^{-1}\right) \end{array}$$

(22) $$\begin{array}{cc} D & =b_{A}I \end{array}$$

(23) $$\begin{array}{cc} g(q) & =-ag\left(\frac{1}{2}m_{a}+m_{b}\right)\left[\begin{array}{c} \sin\left(\phi_{1}\right)\sin\left(\theta_{11}\right)\\ \sin\left(\phi_{2}\right)\sin\left(\theta_{12}\right)\\ \sin\left(\phi_{3}\right)\sin\left(\theta_{13}\right) \end{array}\right]-{\left(J^{T}\right)}^{-1}m\left[\begin{array}{c} 0\\ -g\\ 0 \end{array}\right] \end{array}$$

## 3. Control

### 3.1. Impedance Control

The control proposed for the device is a position and force controller. For this research, an impedance control is applied, which is responsible for producing a reaction force proportional to the variation in the kinetic and potential energy of the mobile with respect to a reference [33]. The controller’s adaptive capability allows the device to adjust its work force automatically. As a result, patient-specific factors, such as age, grip strength, severity of CVD, or time since stroke, will not affect its performance.

The desired effect is a linear combination of elasticity control and damping. To implement the impedance controller, it is necessary to apply the admittance ratio directly [34].

(24) $$\begin{array}{c} \frac{x_{d}\left(s\right)}{F(s)}=\frac{1}{M_{i}s^{2}+B_{i}s+D_{i}}\\ x_{d}\left(s\right)=\frac{F(s)}{M_{i}s^{2}+B_{i}s+D_{i}}\\ \left(M_{i}s^{2}+B_{i}s+D_{i}\right)x_{s}\left(s\right)=F\left(s\right) \end{array}$$

where $x_{d}\left(s\right)$ is the desired reference, F(s)∈R denotes the force applied by the human subject, and $M_{i},\;B_{i},\;D_{i}\in R$ represent the mass, damping and stiffness of the impedance model. In order to adjust the desired position of the device, it is necessary to define the tracking error, which is given by

(25) $$\tilde{x}=x_{d}-x$$

**Figure 10:**
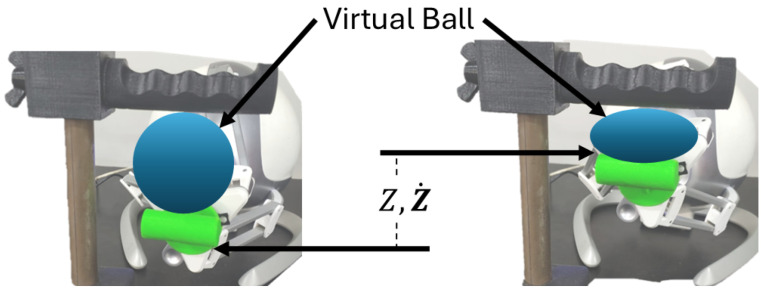
Displacement on Z-axis.

This error is measured with the displacement of the device, as can be seen in Figure 10.

Where *Z* is the vertical position of the end-effector of the Novint Falcon and $\dot{Z}$ is the velocity at which it moves. Therefore, the generalized state vector will have the following form:

(26) $$x=\left[\begin{array}{c} x_{1}\\ x_{2} \end{array}\right]=\left[\begin{array}{c} Z\\ \dot{Z} \end{array}\right]$$

In this way, Equation (24) can be transformed to the time domain, resulting in the following equation:

(27) $$\begin{array}{c} M_{a}\ddot{x}+B_{a}\dot{x}+D_{a}x=f \end{array}$$

where $M_{a},\;B_{a},\;D_{a}\in R$ are positive constants that represents the mass, damping and stiffness of the end-effector, and f is the force applied by the human subject in the end-effector, given by

(28) $$\begin{array}{cc} f=f_{\mathrm{human}}+u\in R & \; \end{array}$$

where $f_{\mathrm{human}}$ is the force applied by the human subject and u is the force applied by the robot. With this in mind, Equation (27) can be rewritten in a matrix form, obtaining

(29) $$\dot{x}=Ax+Bf\;$$

where

(30) $$\begin{array}{c} A=\left[\begin{array}{cc} 0 & 1\\ -\frac{D_{a}}{M_{a}} & -\frac{B_{a}}{M_{a}} \end{array}\right]\;\;B=\left[\begin{array}{c} 0\\ \frac{1}{M_{a}}\; \end{array}\right] \end{array}$$

It can be easily demonstrated that A is a Hurwitz matrix; therefore, (29) is globally exponentially stable.

An adaptive controller on the system can be applied, as expressed in Equation (29). For this, it is necessary to estimate the unknown terms of the model (these estimates are explained later Section 3.2).

(31) $$\dot{\hat{A}}=K_{1}\tilde{x}x_{d}^{T},\;\;\dot{\hat{B}}=K_{2}\tilde{x}f$$

where $K_{1},\;K_{2}\in R^{2\times 2}$ are positive defined diagonal matrices, and f is considered as an approximation of the force applied by the human $f_{\mathrm{human}}$, which is estimated as

(32) $${\hat{f}}_{\mathrm{human}}=\left(\dot{x}-\hat{A}x\right){\hat{B}}^{-1}\;$$

In this way, the control law can be determined by substituting $\dot{x}$ from Equation (32) into Equation (29), resulting in

(33) $$u=\left(-Ax+\hat{A}x+\hat{B}{\hat{f}}_{\mathrm{human}}\right)B^{-1}-{\hat{f}}_{\mathrm{human}}$$

As can be seen, the matrix *A* and the vector *B* form the dynamic model of the system; however, these elements are unknown. For this research, it was decided to approach the issue using a neural network [34,35]. For this case, their approximations are given by $A={\hat{M}}_{A},B={\hat{M}}_{B}$, which are represented as outputs of a single-layer neural network.

(34) $$\begin{array}{c} {\hat{M}}_{A}\left(x,\dot{x}\right)={\hat{W}}_{A}\sigma \left(x,\dot{x}\right),\;M_{A}\left(x,\dot{x}\right)=W_{A}^{*}\sigma \left(x,\dot{x}\right)+\phi \left(x,\dot{x}\right)\\ {\hat{M}}_{B}\left(x,\dot{x}\right)={\hat{W}}_{B}\sigma \left(x,\dot{x}\right),\;M_{B}\left(x,\dot{x}\right)=W_{B}^{*}\sigma \left(x,\dot{x}\right)+\phi \left(x,\dot{x}\right) \end{array}$$

where $W_{A}^{*},\;W_{B}^{*}$ are unknown constant weights, ${\hat{W}}_{A},\;{\hat{W}}_{B}$ are estimated weights, $\phi (x,\dot{x})$ is a neural approximation error, and σ is a function of neuronal activation. The function of this perceptron is to progressively modify its parameters in order to approximate them to the unknown dynamics of the system. This is achieved by adjusting the estimated weights by means of an adaptation law based on the tracking error. The use of a perceptron neural network enables generation of an estimate that is sufficiently acceptable to compensate for the dynamics of the system, reducing possible overadjustments for networks with a greater number of layers [36]. In this way, compensating the dynamics not modeled can be achieved, adding robustness to the control. In this way, the control law is rewritten as follows:

(35) $$u=\left(-{\hat{M}}_{A}x+\hat{A}x+\hat{B}{\hat{f}}_{\mathrm{human}}\right){\hat{M}}_{B}^{-1}-{\hat{f}}_{\mathrm{human}}$$

### 3.2. Stability Proof

**Figure 11:**
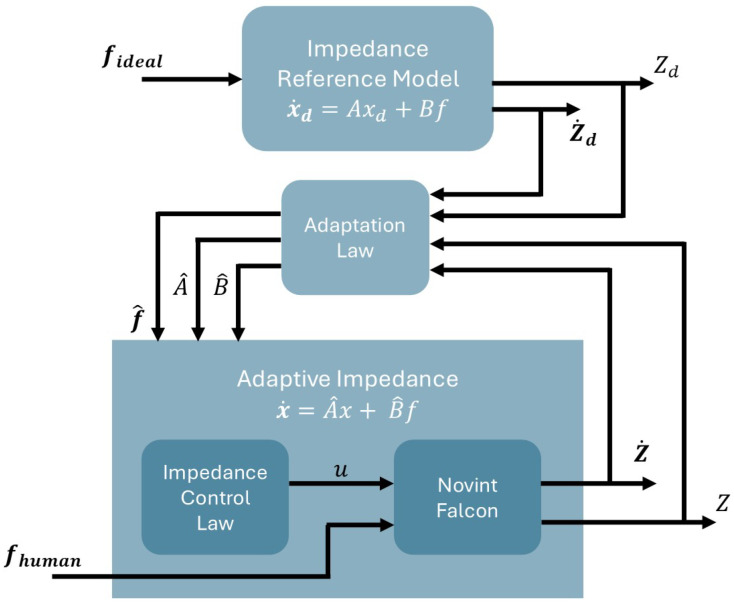
Control block diagram.

Taking Equation (29) as the impedance reference model, whose outputs $Z_{d},\;{\dot{Z}}_{d}$ are the reference trajectories, a variable impedance control law is designed, such that for any input force applied by the human, the robot output will converge to the reference trajectory. Figure 11 represents the block diagram of the control strategy proposed.

Where $x_{d}={\left[Z_{d},\;{\dot{Z}}_{d}\right]}^{T}$ and A, B are unknown, while $\hat{A},\;\hat{B}$ are estimated matrices. The resulting reference model is expressed as

(36) $${\dot{x}}_{d}=Ax_{d}+Bf$$

And the adapted approach as

(37) $$x=\hat{A}x+\hat{B}f\;$$

The arametric errors are defined as

(38) $$\begin{array}{cc} \tilde{A}=A-\hat{A},\; & \;\dot{\tilde{A}}=-\dot{\hat{A}}\\ \tilde{B}=B-\hat{B},\; & \;\dot{\tilde{B}}=-\dot{\hat{B}}\\ \tilde{x}=x_{d}-x,\; & \;\dot{\tilde{x}}={\dot{x}}_{d}-\dot{x} \end{array}\;$$

The parametric error $\dot{\tilde{x}}$ can be rewritten as

(39) $$\begin{array}{cc} \dot{\tilde{x}}= & Ax_{d}+Bf-\hat{A}x-\hat{B}f\\ \dot{\tilde{x}}= & Ax_{d}-\hat{A}\left(x_{d}-\tilde{x}\right)+Bf-\hat{B}f\\ \dot{\tilde{x}}= & Ax_{d}-\hat{A}x_{d}+\hat{A}\tilde{x}+Bf-\hat{B}f \end{array}\;$$

Using Equation (38), the following expression is obtained:

(40) $$\dot{\tilde{x}}=\tilde{A}x_{d}+\hat{A}\tilde{x}+\tilde{B}f\;$$

Continuing with the analysis, the following positive defined function, whose elements are the parametric errors, is proposed as

(41) $$V=\frac{1}{2}{\tilde{x}}^{T}\tilde{x}+\frac{1}{2}tr\left({\tilde{A}}^{T}K_{1}^{-1}\tilde{A}\right)+\frac{1}{2}tr\left({\tilde{B}}^{T}K_{2}^{-1}\tilde{B}\right)$$

Continuing with the demonstration, the derivative is expressed by

(42) $$\begin{array}{cc} \dot{V} & ={\tilde{x}}^{T}\dot{\tilde{x}}+tr\left({\tilde{A}}^{T}K_{1}^{-1}\dot{\tilde{A}}\right)+tr\left({\tilde{B}}^{T}K_{2}^{-1}\dot{\tilde{B}}\right)\\ \dot{V} & ={\tilde{x}}^{T}\left(\tilde{A}x_{d}+\hat{A}\tilde{x}+\tilde{B}f\right)+tr\left({\tilde{A}}^{T}K_{1}^{-1}\dot{\tilde{A}}\right)+tr\left({\tilde{B}}^{T}K_{2}^{-1}\dot{\tilde{B}}\right)\\ \dot{V} & ={\tilde{x}}^{T}\tilde{A}x_{d}+{\tilde{x}}^{T}\hat{A}\tilde{x}+{\tilde{x}}^{T}\tilde{B}f+tr\left({\tilde{A}}^{T}K_{1}^{-1}\dot{\tilde{A}}\right)+tr\left({\tilde{B}}^{T}K_{2}^{-1}\dot{\tilde{B}}\right) \end{array}$$

Using Equation (38), the following expression is obtained:

(43) $$\dot{V}={\tilde{x}}^{T}\tilde{A}x_{d}+{\tilde{x}}^{T}\hat{A}\tilde{x}+{\tilde{x}}^{T}\tilde{B}f-tr\left({\tilde{A}}^{T}K_{1}^{-1}\dot{\hat{A}}\right)-tr\left({\tilde{B}}^{T}K_{2}^{-1}\dot{\hat{B}}\right)\;$$

Considering the following laws of adaptability:

(44) $$\dot{\hat{A}}=K_{1}\tilde{x}x_{d}^{T},\;\;\dot{\hat{B}}=K_{2}\tilde{x}f\;$$

Replacing (44) in (43)

(45) $$\dot{V}={\tilde{x}}^{T}\tilde{A}x_{d}+{\tilde{x}}^{T}\hat{A}\tilde{x}+{\tilde{x}}^{T}\tilde{B}f-tr\left({\tilde{A}}^{T}\tilde{x}x_{d}^{T}\right)-tr\left({\tilde{B}}^{T}\tilde{x}f\right)\;$$

Using the trace property, the following equation is obtained:

(46) $$\begin{array}{cc} \dot{V}= & {\tilde{x}}^{T}\tilde{A}x_{d}+{\tilde{x}}^{T}\hat{A}\tilde{x}+{\tilde{x}}^{T}\tilde{B}f-{\tilde{x}}^{T}\tilde{A}x_{d}-{\tilde{x}}^{T}\tilde{B}f\\ \dot{V}= & {\tilde{x}}^{T}\hat{A}\tilde{x}\leq 0 \end{array}\;$$

Remembering A is a Hurwitz matrix, it can be concluded that the system is semi-definite negative.

By the LaSalle theorem $\dot{V}=0$, then $\tilde{x}\equiv 0$. From (44) and (38), $\tilde{A}\equiv 0$ and $\tilde{B}\equiv 0$, so the origin is asymptotically stable.

## 4. Results

### 4.1. Adaptive Control Simulation

**Figure 12:**
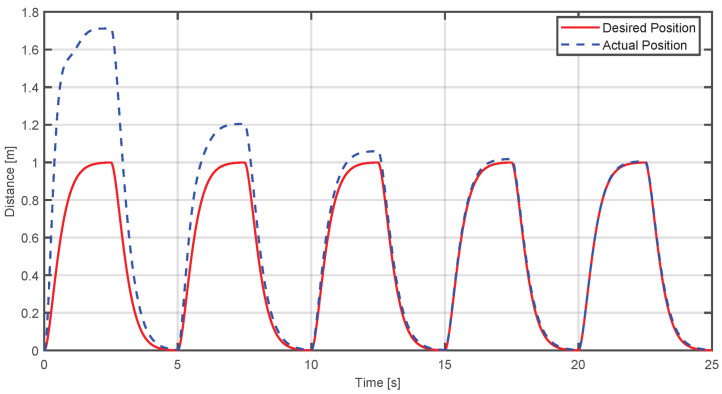
Comparison between the desired and actual position when the applied force exceeds the required level.

An adaptive control simulation was created with MATLAB R2024a, considering the system as a Mass-Spring-Damper. The objective of this simulation is to observe how the adaptive control makes the actual position converge to the desired position; for this case, the reference is a trajectory with over-damped response. In Figure 12, the blue dashed line represents the current position of the end-effector, while the red line is the desired position. It is observed that when the applied force is higher, the actual trajectory initially deviates from the reference. However, the adaptive controller reduces this deviation over time, achieving progressive convergence.

To quantify performance, the Mean Squared Error (MSE) and Mean Absolute Error (MAE) were calculated between the actual and desired trajectories, obtaining the following results:

(47) MSE=0.06007MAE=0.12033

**Figure 13:**
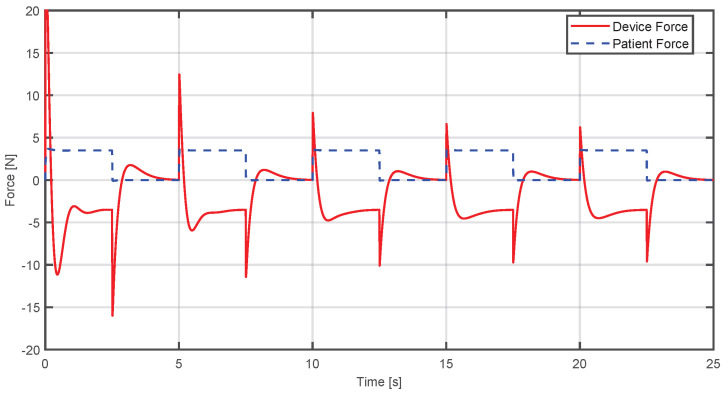
Comparison between the estimated human and device force when the applied force exceeds the required level.

In Figure 13, the red line represents the device’s force, while the blue line is the patient’s simulated force. In this case, the controller increases the device’s force to compensate the user’s force, allowing the trajectory to be followed. This increase generates transient peaks that stabilize at a value close to the patient’s force but in the opposite direction.

**Figure 14:**
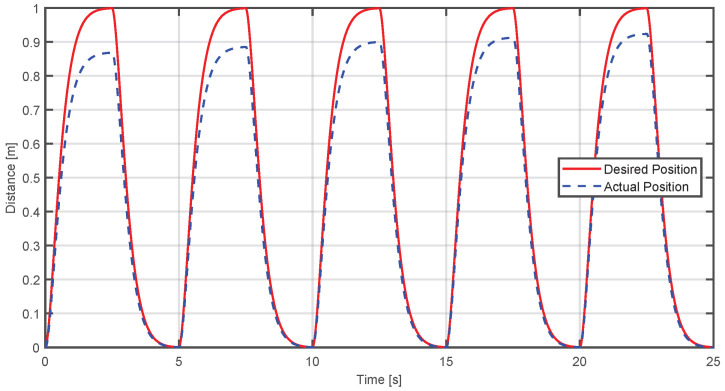
Comparison between the desired and actual position when the applied force is insufficient and the device compensates it.

On the other hand, in Figure 14, the case is shown where the applied force is not sufficient to reach the desired trajectory; the controller reduces the force from the device to achieve convergence to the desired trajectory in a progressive way.

In the same way, to demonstrate a quantitative value, the evaluation metrics MSE and MAE were obtained, whose values are

(48) MSE=0.0045222,MAE=0.052403

**Figure 15:**
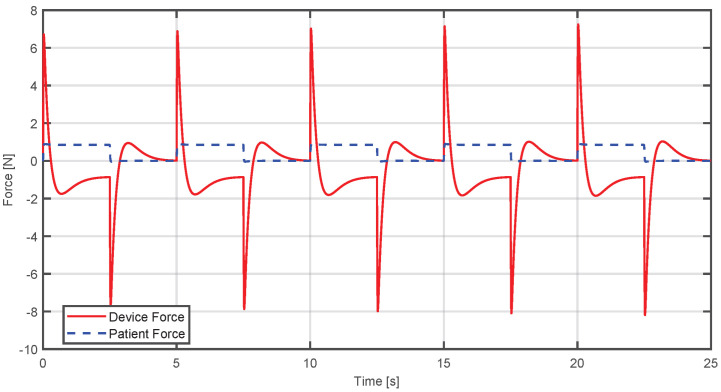
Comparison between the estimated human and device force when the applied force is insufficient and the device compensates it.

In Figure 15, it can be observed that when the user applies a relatively small force, the device will generate an opposite force of comparable magnitude. The aim is to enable the patient to reach the desired trajectory without requiring them to exert more force than they can provide.

### 4.2. Preliminary Tests

**Figure 16:**
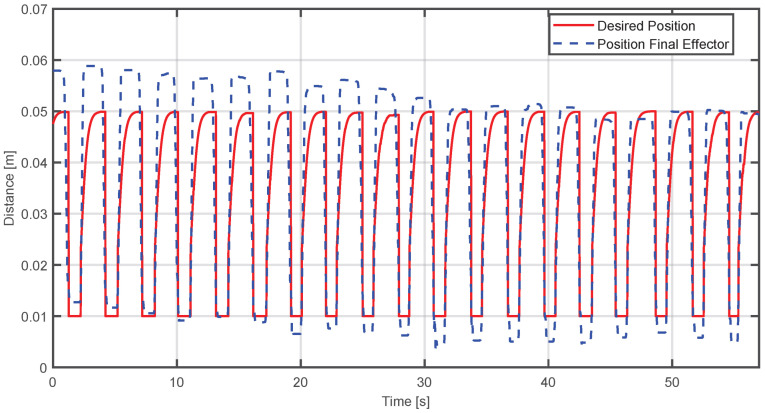
Comparison between the actual position of the end-effector and the desired position when the applied force exceeds the required level.

To check the adaptive control was working, position and force data were collected. For this case, it was decided to work using a sigmoid reference. This trajectory is given by

(49) $$Z_{d}=\frac{0.05}{1+e^{-4(t-0.05)}}$$

where *t* represents the time the step lasted. It was decided to work with this function because an overdamped response from the system was required to avoid abrupt changes that could fatigue the user. Therefore, it is expected that using this type of trajectory will replicate traditional rehabilitation movement. If a more complex trajectory were used, this could affect the natural pattern of the movement, impacting the therapeutic effect. The results presented below show the performance of the adaptive impedance control. In Figure 16, the behavior of the adaptability control is illustrated—when the user applies a force greater than the origin force of the device, the position of the final effector will be above the desired position. As time advances, the adaptive algorithm adjusts the device response, causing the actual position to converge toward the reference.

The evaluation metrics for this case were

(50) $$\mathrm{MSE}=7.7696\times 10^{-05},\;\;\mathrm{MAE}=0.0062496$$

**Figure 17:**
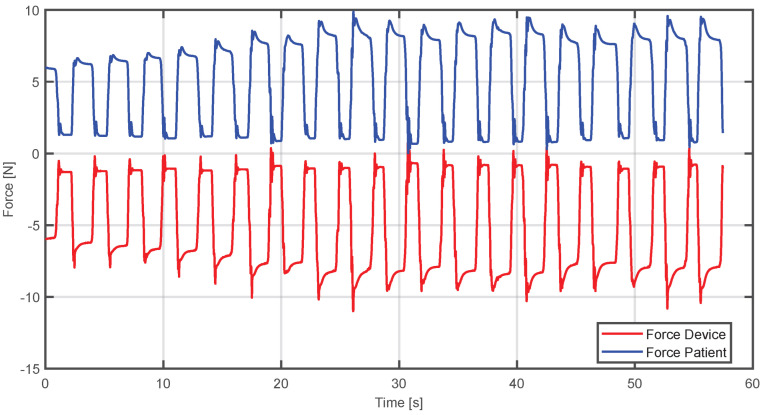
Comparison between estimated human and device forces when the applied force exceeds the required level.

This analysis is supported by Figure 17, where it can be seen that when the patient applies a large force, the device generates an opposite force, until it can be regulated to the point where the forces reach a stable balance, enabling the reference trajectory to be achieved.

**Figure 18:**
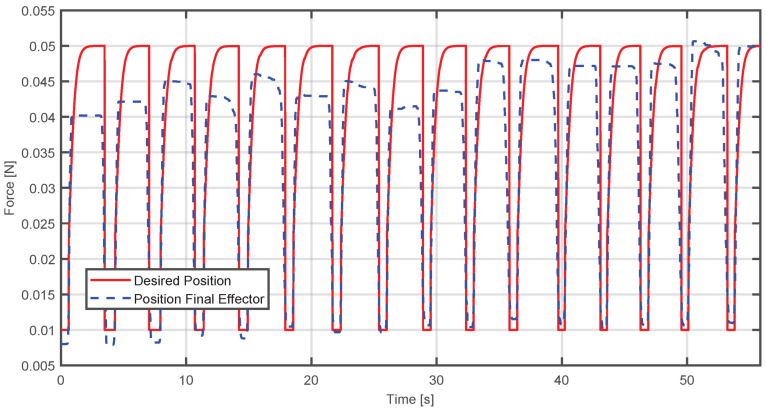
Comparison between the actual position of the end-effector and the desired position when the applied force is insufficient and the device compensates it.

On the other hand, this analysis is complemented by observing the opposite situation. Figure 18 shows that when the patient fails to reach the desired position, the device provides support by generating a lower force proportional to the force of the patient, achieving convergence to the desired trajectory.

The evaluation metrics obtained in this case were

(51) $$\mathrm{MSE}=6.1415\times 10^{-05},\mathrm{MAE}=0.0052469$$

**Figure 19:**
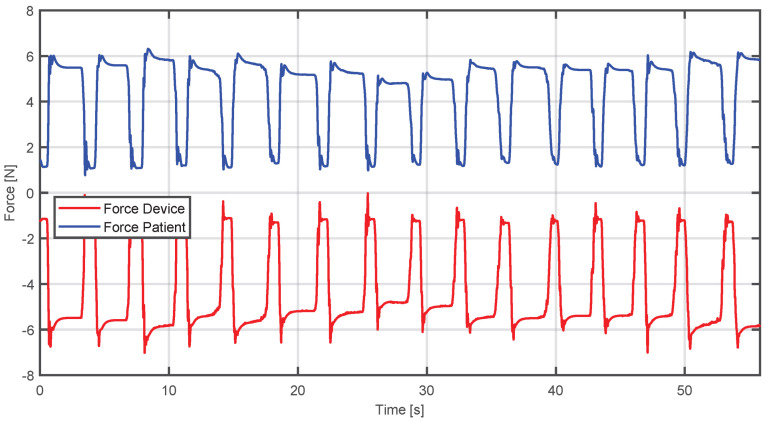
Comparison between estimated human and device forces when the applied force is insufficient and the device compensates it.

In the same way, in Figure 19, it can be seen that the patient’s strength is small compared to Figure 17. Consequently, the device responds with a lower and opposite force to guide the system to the desired position without imposing an excessive demand on the patient.

### 4.3. Experimental Results

Tests were performed with 10 volunteers, of which eight were men and two were women between the ages of 21 to 32. These participants did not present with any condition associated with muscle weakening in the hand. These tests were designed only to validate the performance of the device. The game with which the rehabilitation was to be performed was modified to compel the users to lose when reaching a time between 1 and 2 min.

In order to perform the rehabilitation exercise correctly, the patient required to be seated in front of the haptic device, placed such that the end-effector and the handle are at a suitable height for the patient’s hand. Once the patient is in a comfortable position, registration occurs, followed by positioning of the final effector at the origin, waiting for the game to begin. When the game is running, the patient must perform a pinch movement in response to visual stimuli, represented by means of obstacles that the patient must avoid. With each movement of the user, the device is responsible for adapting the force necessary to remain similar to the force applied by the patient. If the patient impacts with an obstacle, the game automatically ends. A final window is shown that presents information on the time and obstacles overcome. If the volunteers express pain, fatigue or discomfort, the session would end to adjust the strength of the device.

Once the session is over, the patient data are saved. The data collected for some participants are shown in Table 1. This database is planned to be used with patients in order to monitor any progress in their rehabilitation. Here, only sessions in which they performed best are presented.

**Figure 20:**
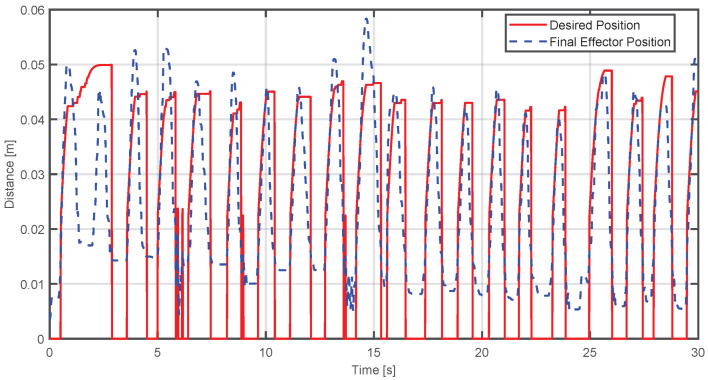
Comparison between the actual position of the end-effector and the desired position during virtual environment execution.

**Figure 21:**
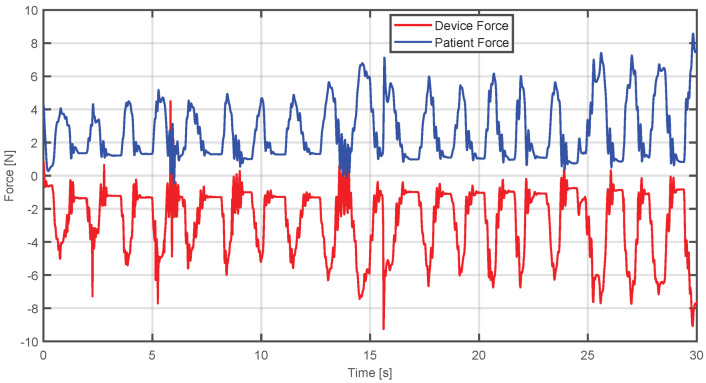
Comparison between the estimated human and device forces during virtual environment execution.

Figure 20 presents the behavior of the controller of a single participant. One thing that should be highlighted is that the desired position does not manage to complete in most cases. This is due to the configuration of the virtual environment where the movement to be developed can become faster because it is working with people without any condition. However, it can still be seen that the position of the effector begins to converge at the maximum position of the sigmoid function used as the desired position:

In the same way, Figure 21 shows how the force of the device is compensated according to the force applied by the participant in each of their movements:

The metrics obtained for this case were

(52) MSE=0.00015884,MAE=0.010122

## 5. Conclusions

This research proposed a rehabilitation exercise using a variable impedance controller. A key advantage of this system is its ability to obtain relevant measurements for clinical monitoring [37]. Furthermore, its haptic and force-adaptive capabilities are crucial; the device can deliver specific forces based on the situation and adjust to any user, regardless of their initial strength.

However, the system has limitations. Notably, the Novint Falcon has a limited maximum force, which could cause the device to no longer pose a challenge for patients nearing recovery or in advanced stages of rehabilitation. Another drawback is the initial adaptation time, which can be slow. Nevertheless, once the force has adapted, it remains constant throughout the session.

The exercise focused on the pinch movement to rehabilitate the grip force. The results show three different cases. In the first one, a simulation of the controller against a constant simulated human force showed that the system behaved as anticipated. In the second case, the variable impedance controller was tested on the Novint Falcon device. These tests demonstrated successful force adaptation along the desired trajectory in two scenarios; the device increased its opposing force when the user’s force was higher than the target, and it reduced its force when the user’s input was lower.

Finally, the exercise was tested on 10 healthy volunteers with no grip strength impairments. This stage was designed to demonstrate the functionality of the haptic device and virtual environment, not for clinical validation. The results showed that the desired sigmoidal path was not followed perfectly; however, the graphical data indicate that the end-effector’s position consistently attempted to converge on the desired trajectory. For example, Figure 20 shows that it takes approximately 10 to 15 s for the system to begin following the desired trajectory. This delay is the time the device needs to adapt to the user’s force. While this period may seem long, it is not considered problematic within a typical 30 min rehabilitation session.

Looking ahead to clinical application, it is important to note that the speed and difficulty of the video game will be adjusted to each patient’s physical abilities. This will allow for personalized sessions that can be extended as needed, ensuring a tailored rehabilitation experience.

## Figures and Tables

**Table 1:** Data obtained from tests.

Patient_id	01	02	03	04	05
Username	S01	S02	S03	S04	S05
Session_number	07	06	04	03	04
Play_time (s)	132.94	119.104	107.178	95.6022	156.702
Pipes_passed	89	73	61	50	119
Movement_count	103	95	98	83	127
Average_force (N)	5.32048	4.3042	2.209807	2.20932	4.1819
Session_date	7 July 2025	7 July 2025	7 July 2025	7 July 2025	7 July 2025
Patient_id	06	07	08	09	10
Username	S06	S07	S08	S09	S10
Session_number	07	06	04	03	04
Play_time (s)	30.1914	61.5911	70.438	146.008	87.4134
Pipes_passed	8	24	30	105	43
Movement_count	21	49	54	128	72
Average_force (N)	3.29855	2.36627	1.50723	2.68128	2.34491
Session_date	7 July 2025	8 July 2025	8 July 2025	8 July 2025	8 July 2025

## Data Availability

Data are contained within the article.
